# Effects of anti-VEGF therapy on choroidal thickness in eyes with diabetic macular edema

**DOI:** 10.1186/s12886-026-04811-y

**Published:** 2026-04-10

**Authors:** Xiao-nan Shi, Qian-ying Zhang, Chao-juan Ju, Yin-cong Xu, Zhao-hui Xiong

**Affiliations:** Department of Ophthalmology, The First Hospital of Hebei Medical University, Shijiazhuang, Hebei 050000 China

**Keywords:** Diabetic macular edema, Choroidal thickness, Anti-VEGF, Conbercept, Optical coherence tomography

## Abstract

**Background:**

Diabetic macular edema (DME) is a leading cause of visual impairment in working-age adults. Although anti-vascular endothelial growth factor (VEGF) therapy is the standard treatment for DME, its effects on choroidal structure remain incompletely characterized. This study aimed to evaluate changes in subfoveal choroidal thickness (SFCT) following intravitreal Conbercept injection in eyes with center-involved DME.

**Methods:**

In this prospective study, 40 patients (51 eyes) with center-involved DME were enrolled between August 2019 and November 2022. Based on diabetic retinopathy severity, eyes were classified into non-proliferative DR with macular edema (NPDR-ME, n = 36) and proliferative DR with macular edema (PDR-ME, n = 15) groups. All eyes received intravitreal Conbercept (0.5 mg) injections. Best-corrected visual acuity (BCVA), central macular thickness (CMT), and SFCT were measured using spectral-domain optical coherence tomography at baseline and at 1, 3, and 6 months post-treatment. Repeated measures ANOVA and Spearman correlation analyses were performed.To account for the correlation between paired eyes from patients with bilateral involvement, data were analyzed using generalized estimating equations (GEE).

**Results:**

At 6 months post-treatment, significant improvements were observed in logMAR BCVA (0.61 ± 0.25 to 0.33 ± 0.23, P < 0.001) and CMT (466.25 ± 129.85 to 279.41 ± 72.47 μm, P < 0.001). SFCT decreased progressively from 379.65 ± 60.59 μm at baseline to 304.63 ± 53.92 μm at 6 months (P < 0.001). No significant differences in SFCT were observed between NPDR-ME and PDR-ME groups at any time point (P > 0.05). BCVA at 6 months correlated significantly with baseline and post-treatment BCVA and CMT (P < 0.05) but not with SFCT at any time point (P > 0.05).

**Conclusion:**

Intravitreal Conbercept significantly reduces subfoveal choroidal thickness in DME eyes, accompanied by improved visual acuity and reduced macular edema. The lack of correlation between SFCT changes and visual outcomes suggests that choroidal thickness may not directly reflect functional improvement.:

## Background

Diabetic retinopathy (DR) remains a leading cause of preventable blindness among working-age adults worldwide, with an estimated 366 million people affected by diabetes by 2030 [1, 2]. Diabetic macular edema (DME), characterized by fluid accumulation in the macula due to breakdown of the blood-retinal barrier, represents the most common cause of visual impairment in patients with DR and can occur at any stage of the disease [1, 3].

The pathogenesis of DME involves complex interactions between metabolic, inflammatory, and vascular factors. Chronic hyperglycemia induces oxidative stress, accumulation of advanced glycation end-products, and upregulation of pro-inflammatory cytokines, ultimately leading to increased vascular endothelial growth factor (VEGF) expression [4, 5]. VEGF disrupts endothelial tight junctions and increases vascular permeability, contributing to macular edema. While the role of retinal vascular changes in DME is well-established, increasing evidence suggests that choroidal dysfunction may also contribute to disease pathogenesis [6].

The choroid supplies approximately 85% of ocular blood flow and is responsible for nourishing the outer retina, including photoreceptors and the retinal pigment epithelium (RPE) [7]. Structural and functional alterations in the choroidal vasculature may therefore influence retinal health and the development of macular edema. Subfoveal choroidal thickness (SFCT), measured using enhanced depth imaging optical coherence tomography (EDI-OCT), serves as a surrogate marker for choroidal blood flow and vascular status [7]. Previous studies have reported conflicting findings regarding choroidal thickness in DME, with some showing increased thickness attributed to inflammation and VEGF-mediated vasodilation [8, 9], while others demonstrate thinning due to microvascular dropout and ischemia [10–12].

Anti-VEGF therapy has revolutionized DME management, with multiple randomized controlled trials demonstrating efficacy in improving visual acuity and reducing retinal thickness [13, 14]. However, the effects of anti-VEGF on choroidal structure remain incompletely understood. Several studies have reported choroidal thinning following anti-VEGF treatment in DME [15, 16], but the mechanisms underlying this phenomenon and its relationship with functional outcomes require further investigation. Moreover, whether choroidal thickness changes differ according to DR severity (NPDR vs. PDR) and whether baseline choroidal thickness predicts treatment response remain unclear.

Therefore, this prospective study aimed to: (1) quantify changes in SFCT following intravitreal Conbercept injection in eyes with center-involved DME; (2) compare choroidal thickness changes between NPDR-ME and PDR-ME groups; (3) examine the correlation between SFCT changes and visual acuity improvement; and (4) explore potential mechanisms underlying anti-VEGF-induced choroidal thinning.

### Subjects and clinical study protocol

This prospective, single-center, interventional study was conducted at the Department of Ophthalmology, The First Hospital of Hebei Medical University, Shijiazhuang, China, between August 2019 and November 2022. The study protocol was approved by the Institutional Ethics Committee (Approval No. 20200646) and adhered to the tenets of the Declaration of Helsinki. Written informed consent was obtained from all participants after explanation of the study purpose and procedures.

A total of 40 participants (51 eyes) diagnosed with center-involved diabetic macular edema (DME) at the Department of Ophthalmology, the First Hospital of Hebei Medical University, from August 2019 to November 2022 were enrolled in this study. Among them, 16 were male participants (21 eyes) and 24 were female participants (30 eyes), with a mean age of (56.45 ± 10.35) years. The duration of macular edema before treatment ranged from 5 days to 1 month. Among the 51 eyes, 36 eyes (70.6%, 36/51) were complicated with non-proliferative diabetic retinopathy (NPDR), assigned to the NPDR-ME group; and 15 eyes (29.4%, 15/51) were complicated with proliferative diabetic retinopathy (PDR), assigned to the PDR-ME group.

Diabetic retinopathy severity was graded according to the modified Early Treatment Diabetic Retinopathy Study (ETDRS) severity scale based on fundus fluorescein angiography (FFA) findings. Eyes were classified into two groups: (1) NPDR-ME group: eyes with non-proliferative diabetic retinopathy (microaneurysms, retinal hemorrhages, hard exudates, cotton-wool spots, or venous beading without evidence of neovascularization); (2) PDR-ME group: eyes with proliferative diabetic retinopathy (neovascularization of the disc [NVD], neovascularization elsewhere [NVE], vitreous hemorrhage, or preretinal fibrosis). Grading was performed by two independent retina specialists masked to clinical data; discrepancies were resolved by consensus.

### Inclusion criteria

(1) diagnosis of type 1 or type 2 diabetes mellitus confirmed by the Department of Endocrinology; (2) age 30–80 years; (3)Meeting the diagnostic criteria for diabetic macular edema (DME) [17]; (4) well-controlled blood glucose (fasting glucose ≤ 6.1 mmol/L and postprandial glucose ≤ 11.1 mmol/L) on stable anti-diabetic medication for at least 3 months.

### Exclusion criteria

(1) refractive error exceeding ± 3.0 diopters (spherical equivalent); (2) significant media opacity pretaining acquisition of high-quality OCT images; (3) intraocular pressure > 21 mmHg or diagnosis of glaucoma; (4) concurrent retinal or choroidal pathology including age-related macular degeneration, retinal vein occlusion, polypoidal choroidal vasculopathy, uveitis, or macular telangiectasia; (5) history of focal/grid laser photocoagulation, panretinal photocoagulation (PRP), vitrectomy, intravitreal injections, or any other intraocular surgery; (6) evidence of ischemic maculopathy on fundus fluorescein angiography (FFA), defined as foveal avascular zone (FAZ) enlargement or capillary dropout exceeding 1 disc area; (7) uncontrolled systemic hypertension (systolic > 160 mmHg or diastolic > 100 mmHg) or other endocrine disorders; (8) pregnancy or lactation; (9) known allergy to fluorescein or anti-VEGF.

## Methods

All participants underwent comprehensive ophthalmic examination at baseline and at 1, 3, and 6 months post-treatment, including:

Best-corrected visual acuity (BCVA): Measured using the ETDRS visual acuity chart at 4 m under standardized lighting conditions. BCVA was recorded as the total number of letters read correctly and converted to logMAR units for statistical analysis.

Optical coherence tomography (OCT): All images were acquired using the Cirrus HD-OCT 5000 device (Carl Zeiss AG, Germany) with the enhanced depth imaging (EDI) mode. A 6 mm horizontal and vertical line scan centered on the fovea was performed, covering a 6 × 6 mm area. Only images with signal strength ≥ 7/10 and quality index > 50 were included. Central macular thickness (CMT) was defined as the average retinal thickness in the central 1 mm subfield of the ETDRS grid, measured from the internal limiting membrane to the outer border of the retinal pigment epithelium (RPE). Subfoveal choroidal thickness (SFCT) was defined as the vertical distance from the outer border of the hyperreflective RPE line to the inner surface of the sclera, measured manually at the foveal center using the caliper function. All measurements were performed by the same experienced ophthalmologist who was masked to clinical data and time point. Three measurements were obtained for each parameter, and the average value was used for analysis. Intraobserver reproducibility was assessed using intraclass correlation coefficient (ICC), which was 0.97 (95% CI: 0.95–0.98) for CMT and 0.94 (95% CI: 0.91–0.96) for SFCT.

Fundus fluorescein angiography (FFA): FFA was performed at baseline using the Spectralis HRA + OCT device (Heidelberg Engineering, Germany). After intravenous injection of 5 mL of 10% sodium fluorescein, early-phase (1–2 min) and late-phase (5–10 min) images were acquired to assess macular ischemia, capillary non-perfusion, and neovascularization. Eyes with evidence of ischemic maculopathy (FAZ enlargement or capillary dropout) were excluded.

### Treatment protocol

All intravitreal injections were performed under strict aseptic conditions in a laminar flow operating room. Following topical anesthesia with 0.5% proparacaine hydrochloride and povidone-iodine disinfection of the conjunctival sac and eyelid margins, 0.05 mL of Conbercept solution (10 mg/mL, containing 0.5 mg Conbercept; Chengdu Kanghong Biotech Co., Ltd., China) was injected into the vitreous cavity using a 30-gauge needle inserted 3.5–4.0 mm posterior to the limbus in the inferotemporal quadrant. All injections were administered by a single experienced retina specialist.

A 3 + PRN treatment regimen was applied. This consisted of three consecutive monthly loading doses, followed by additional injections as needed, administered by the same physician based on clinical evaluation.

Follow-up visits were scheduled at 1, 3, and 6 months after the initial injection. At each visit, all participants underwent BCVA measurement, intraocular pressure measurement, slit-lamp examination, and OCT imaging using identical protocols as baseline. The number of injections received during the follow-up period was recorded, along with any treatment-related complications (transient intraocular pressure elevation, endophthalmitis, cataract progression, neovascular glaucoma, or systemic adverse events).

Considering that some patients had bilateral eyes included in this prospective study, generalized estimating equations (GEE) were applied to analyze the data to account for the correlation between paired eyes from the same patient, thus avoiding treating fellow eyes as independent observations. Statistical analysis was performed using SPSS 27.0 software. Measurement data were expressed as mean ± standard deviation (x ± s); differences were compared using ANOVA or t-tests. Enumeration data were expressed as frequency and composition ratio [N (%)]; differences were compared using χ² tests. Repeated Measures ANOVA was used to analyze changes in indicators across different time points, while pairwise comparisons between time points were performed using the Least Significant Difference t-test. The correlation between BCVA and various indicators at 6 months post-treatment was analyzed using the Spearman test. The significance level was set at α = 0.05. A P-value < 0.05 was considered statistically significant.

## Results

At 6 months post-treatment, the mean number of injections was (3.6667 ± 0.89974) in the PDR group and (3.3056 ± 1.11661) in the NPDR group. No statistically significant difference was observed in the number of injections between the two groups (t = -1.109, *P* > 0.05).

Table 1 shows changes in logMAR BCVA, CMT, and SFCT at baseline and 1, 3, 6 months post-treatment. Compared with baseline, all parameters improved significantly at each follow-up time point (all *P* < 0.001)

**Table 1:** Changes in visual acuity, retinal thickness and choroidal thickness of the DME group at different time points

Time Point	Number of Eyes	logMAR BCVA	CMT(µm)	SFCT(µm)
Baseline	51	0.61 ± 0.25	466.25 ± 129.85	379.65 ± 60.59
1 Month Post-treatment	51	0.46 ± 0.26	341.16 ± 99.67	339.55 ± 54.73
3 Months Post-treatment	51	0.40 ± 0.27	300.80 ± 101.59	321.14 ± 61.10
6 Months Post-treatment	51	0.33 ± 0.23	279.41 ± 72.47	304.63 ± 53.92
F value	—	77.778	170.414	141.334
P value	—	<0.001	<0.001	<0.001

Both the PDR-ME and NPDR-ME groups showed significant improvements in BCVA and reductions in CMT and SFCT at 1, 3, and 6 months post-treatment compared with baseline (all *P* < 0.001; Table 2).

**Table 2:** Changes in visual acuity, retinal thickness, and choroidal thickness at different time points in the PDR-ME and NPDR-ME groups

Time Point	PDR-ME Group (*n* = 15)			NPDR-ME Group (*n* = 36)		
	logMAR BCVA	CMT(µm)	SFCT(µm)	logMAR BCVA	CMT(µm)	SFCT(µm)
Baseline	0.727 ± 0.2764	534.8 ± 116.238	374.00 ± 75.263	0.564 ± 0.2295	429.36 ± 96.472	382.00 ± 54.403
1 Month Post-treatment	0.593 ± 0.2915	346.67 ± 108.821	319.73 ± 60.155	0.4 ± 0.2318	338.86 ± 97.141	347.81 ± 50.923
3 Months Post-treatment	0.5 ± 0.3071	291 ± 63.32	313.07 ± 27.468	0.356 ± 0.2501	277.11 ± 53.197	310.89 ± 37.087
6 Months Post-treatment	0.44 ± 0.2947	259.73 ± 45.01	289.4 ± 39.614	0.2792 ± 0.18102	257.06 ± 41.313	294.31 ± 36.965
F	34.845	72.182	18.410	46.411	122.518	159.732
p	<0.001	<0.001	<0.001	<0.001	<0.001	<0.001

SFCT decreased significantly in the overall DME group at each post-treatment time point (*P* < 0.001). There were no significant between-group differences in SFCT at any time point (*P* > 0.05; Table 3).

**Table 3:** Changes in choroidal thickness of the PDR-ME group and NPDR-ME group at different time points post-treatment

Group	SFCT(µm) (1 Month )	SFCT(µm) ( 3 Month )	SFCT(µm)( 6 Month )
PDR-ME group	319.73 ± 60.155	313.07 ± 27.468	289.4 ± 39.614
NPDR-ME group	347.81 ± 50.923	310.89 ± 37.087	294.31 ± 36.965
t	-1.433	1.268	-0.355
P	>0.05	>0.05	>0.05

Results of Spearman’s rank correlation analysis showed that the best-corrected visual acuity (BCVA) at 6 months post-treatment was significantly correlated with the BCVA at baseline, 1 month and 3 months post-treatment, as well as the central macular thickness (CMT) values at baseline and 1, 3, 6 months post-treatment (*r* = 0.690, 0.852, 0.862, 0.530, 0.442, 0.385, 0.322; all *P* < 0.05). However, no significant correlation was found between the BCVA at 6 months post-treatment and the subfoveal choroidal thickness (SFCT) values at baseline and 1, 3, 6 months post-treatment (*R* = 0.006, 0.053, 0.124, 0.05; all *P* > 0.05).

During the follow-up period, none of the affected eyes developed complications such as transient intraocular pressure elevation, endophthalmitis, cataract progression, or neovascular glaucoma.

**Fig. 1:**
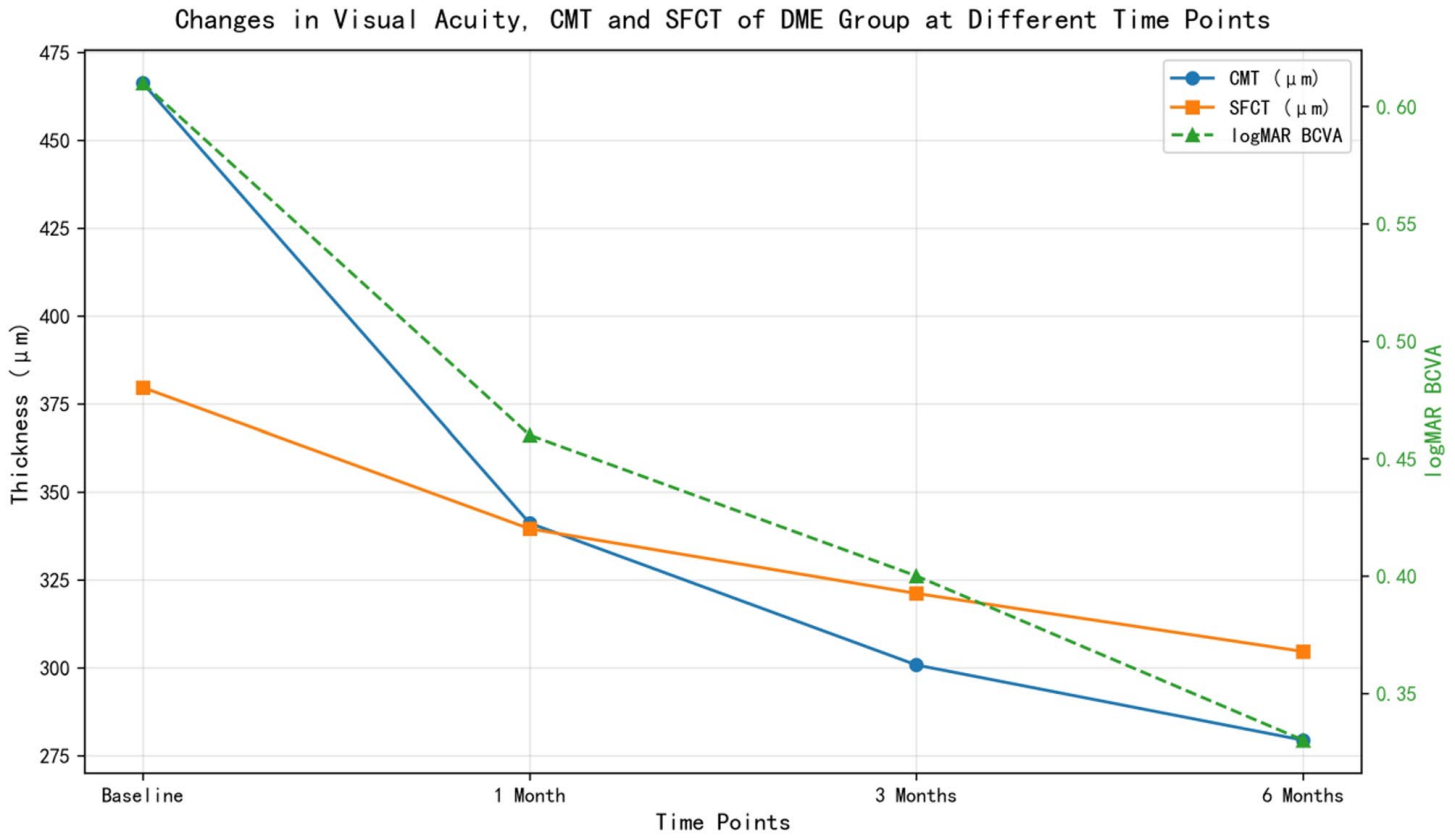
Changes in logMAR BCVA, CMT and SFCT of the DME group before and post-treatment

**Fig. 2:**
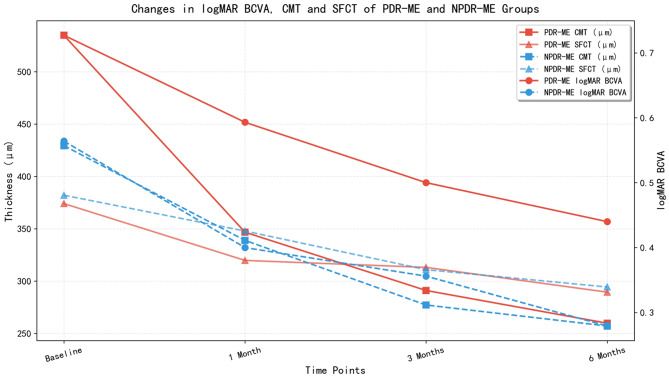
Changes in logMAR BCVA, CMT and SFCT of PDR-ME and NPDR-ME groups before and post-treatment

**Fig. 3:**
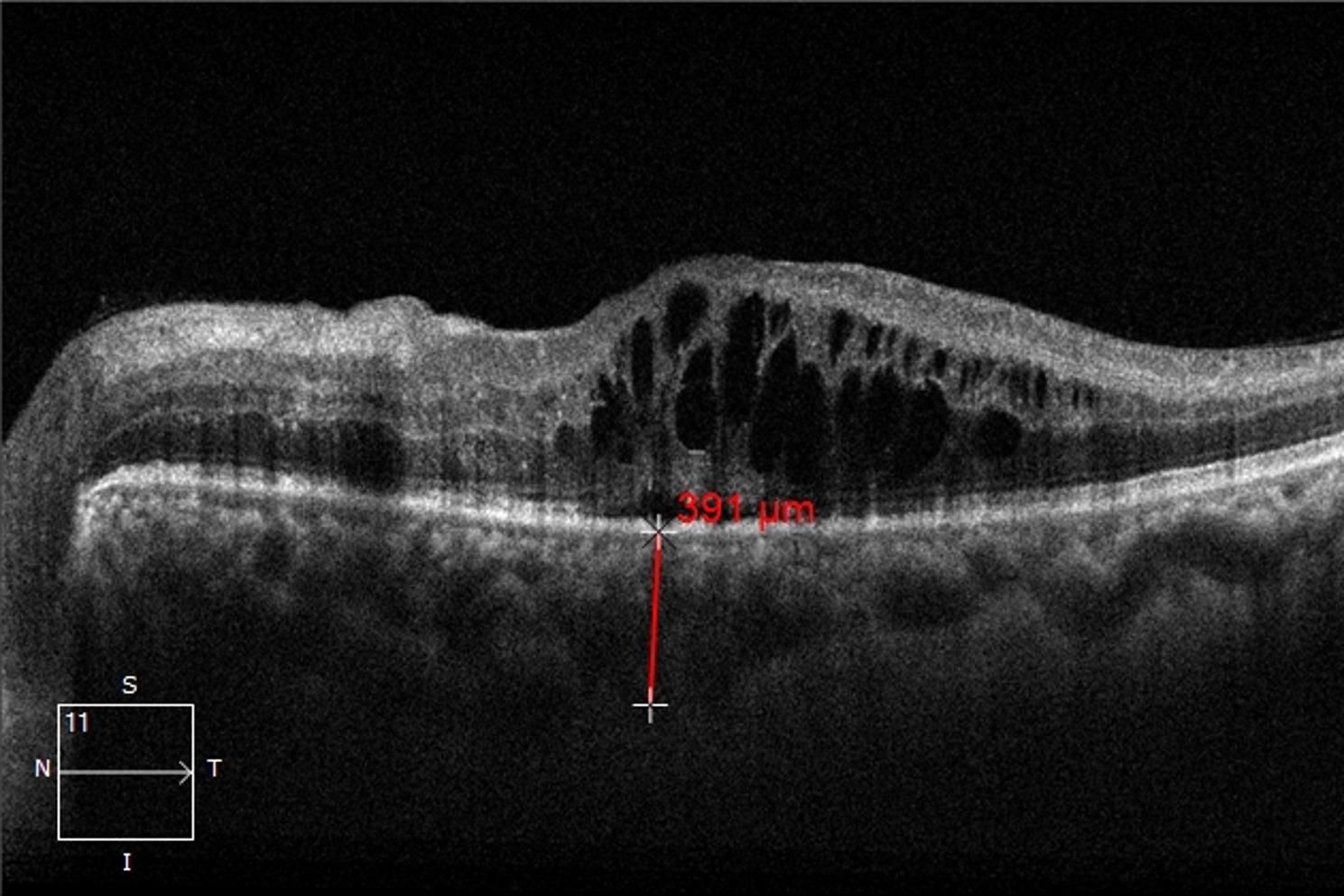
Representative EDI-OCT image at baseline in a patient with DME. Cystoid macular edema and subretinal fluid are present. Subfoveal choroidal thickness (SFCT) was measured from the outer border of the RPE to the inner scleral surface at the fovea, with a value of 391 μm

**Fig. 4:**
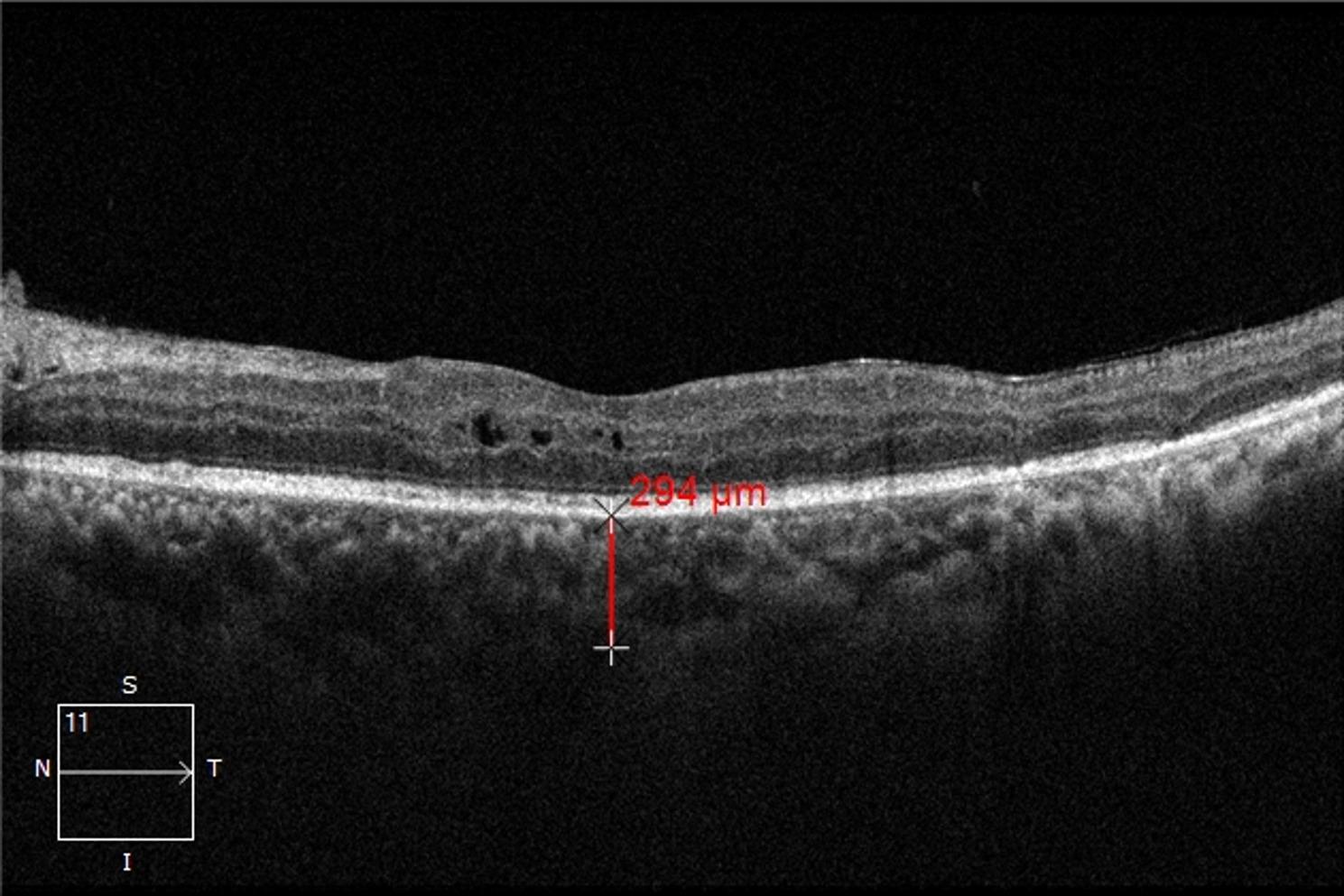
Representative EDI-OCT image at 6 months post-treatment in the same patient. Cystoid macular edema has resolved and subretinal fluid has been absorbed. SFCT at the fovea is reduced to 294 μm, consistent with anti-VEGF-induced choroidal thinning

## Discussion

Vascular endothelial growth factor (VEGF) disrupts the blood-retinal barrier, leading to vascular leakage and diabetic macular edema (DME) [5]. anti-VEGF are currently the first-line treatment for DME worldwide [13]. In this prospective study, we investigated the effects of intravitreal Conbercept injection on subfoveal choroidal thickness (SFCT) in patients with DME. The main findings revealed that: (1) anti-VEGF therapy significantly reduced SFCT at 1, 3, and 6 months post-treatment; (2) central macular thickness (CMT) decreased and best-corrected visual acuity (BCVA) improved significantly; and (3) no significant correlation was observed between SFCT changes and visual acuity improvement. These findings are consistent with previous studies reporting choroidal thinning following anti-VEGF treatment in DME patients [15, 16, 18].

### Choroidal structure and function in DME

The choroid, primarily composed of blood vessels derived from the short posterior ciliary arteries, supplies approximately 85% of total ocular blood flow [7]. Subfoveal choroidal thickness (SFCT) serves as a surrogate marker for choroidal blood flow and reflects the metabolic and functional status of retinal tissue [19]. Structural and functional alterations in the choroidal vasculature may therefore influence retinal health and the development of macular edema [6].

In our cohort, baseline choroidal thickness (379.65 ± 60.59 μm) was slightly higher than that reported in some studies [10–12], a finding consistent with previous reports suggesting that DME eyes may exhibit increased choroidal thickness prior to treatment [8, 9]. The causes of increased choroidal thickness may be associated with inflammatory responses and elevated VEGF levels. Elevated VEGF not only increases retinal vascular permeability but also enhances choroidal vessel permeability [20]. Excessive permeability and dilation of choroidal vessels are primary contributors to increased choroidal thickness [21]. Meanwhile, elevated VEGF levels promote nitric oxide (NO) production, which may also lead to choroidal vasodilation and thickening [22].

Recent evidence confirms that inflammatory responses play a crucial role in diabetic retinopathy (DR) pathogenesis. Inflammatory factors such as C-reactive protein, interleukin-1β (IL-1β), and tumor necrosis factor-α (TNF-α) are closely correlated with DR progression [23, 24]. Campos et al. [8, 9] demonstrated in a diabetic rat models that choroidal thickness increases in early diabetes due to inflammatory cell infiltration and VEGF upregulation, while vascular density paradoxically decreases. These findings support the notion that choroidal thickening in DME reflects inflammatory edema of the choroidal stroma rather than true vascular proliferation, which may explain why anti-VEGF therapy reduces choroidal thickness by suppressing VEGF-mediated vascular permeability and inflammation.

### Comparison with existing literature

The observed reduction in SFCT following anti-VEGF therapy aligns with findings from Western populations. Vinicius et al. [15] reported significant choroidal thinning in DME patients three months after ranibizumab injection, while Laíns et al. [16] demonstrated similar effects with bevacizumab. It has been reported that anti-VEGF treatment induces choroidal thinning not only in DME-affected eyes but also in eyes with other retinal vascular diseases, such as retinal vein occlusion and age-related macular degeneration [25, 26].

Interestingly, no significant difference in SFCT was observed between the NPDR-ME and PDR-ME groups at any time point before or post-treatment—a finding that contrasts with previous reports suggesting that choroidal thickness varies with DR severity [27–29]. Sabour et al. [27] demonstrated that choroidal thickness gradually decreases during progression from non-DR to NPDR, whereas it increases during transition from NPDR to PDR. Similarly, Shen et al. [29] reported that SFCT in diabetic patients is thinner than in healthy individuals and increases as DR severity progresses.

Several factors may explain this discrepancy. First, our study population consisted exclusively of DME patients, whereas previous studies included DR patients without macular edema. The presence of macular edema itself may alter choroidal hemodynamics, potentially masking severity-related differences. Second, the relatively small sample size, particularly in the PDR-ME group (*n* = 15), may have limited statistical power to detect between-group differences. Third, all patients in our study had center-involved DME, which may represent a more advanced stage of macular pathology where DR severity exerts less influence on choroidal structure. Although some participants were assigned to the NPDR group based on FFA findings, the pathological changes in their macular region may have been similar to those in the PDR group due to the presence of macular edema.

### Potential mechanisms underlying choroidal thinning

The reduction in SFCT following anti-VEGF therapy may involve multiple mechanisms. First, VEGF is known to increase vascular permeability and induce vasodilation through NO production [22]. Intravitreal anti-VEGF can penetrate the retina and reach the choroid via diffusion through the retinal pigment epithelium (RPE) or through systemic absorption following entry into the circulation [25]. Once in the choroid, these agents inhibit VEGF-mediated vascular permeability, reduce choroidal vessel dilation, and decrease extravascular fluid accumulation, leading to reduced choroidal thickness.

Second, anti-VEGF may induce choroidal vasoconstriction by inhibiting VEGF’s role as a survival factor for endothelial cells and its vasodilatory effects mediated by NO [22]. Studies have shown that VEGF blockade reduces choroidal blood flow, which may contribute to thickness reduction [30]. Third, the mechanical effect of intravitreal injection itself may transiently alter ocular perfusion pressure and choroidal blood flow, although this effect is likely short-lived [30].

The choroidal thinning observed in our study may reflect structural changes at the microvascular level. Recent advances in optical coherence tomography angiography (OCTA) have enabled more detailed characterization of choroidal vascular changes in diabetes. Borrelli et al. [6] demonstrated that diabetic patients exhibit reduced choroidal vascular density even in the absence of clinical retinopathy, suggesting that choroidal involvement occurs early in the disease process.

### Relationship between choroidal thickness and visual outcomes

The lack of correlation between SFCT changes and visual acuity improvement (*R* = 0.006–0.124, *P* > 0.05) suggests that choroidal thickness may not directly reflect functional outcomes. Visual acuity in DME is primarily determined by foveal structural integrity, including resolution of intraretinal fluid and restoration of photoreceptor function. Choroidal thickness changes may represent a secondary phenomenon related to VEGF suppression rather than a direct determinant of visual outcomes. This interpretation is supported by Rayess et al. [26], who found that baseline choroidal thickness did not predict visual acuity improvement following anti-VEGF therapy. Although studies have suggested that baseline choroidal thickness may serve as a biomarker for disease severity and prognostic prediction [31], our findings indicate that short-term changes in SFCT do not correlate with visual recovery, which may be related to the relatively short follow-up duration.

### Clinical implications

The finding that anti-VEGF therapy reduces choroidal thickness may have clinical relevance for monitoring treatment response. While CMT remains the primary anatomical outcome measure, SFCT may serve as an additional biomarker reflecting the pharmacological effect of VEGF suppression on the choroidal vasculature. However, the absence of correlation with visual acuity suggests that SFCT should not be used as a surrogate for functional outcomes.

### Limitations

Several limitations should be acknowledged. First, the absence of a control group and the relatively small sample size—particularly in the PDR-ME subgroup (*n* = 15)—limit our ability to definitively attribute observed changes to anti-VEGF therapy rather than natural disease progression, and may have reduced statistical power to detect between-group differences. Second, the inclusion of both eyes from some patients (51 eyes from 40 patients) created statistical dependency; although this was not addressed in the primary analysis, sensitivity analyses using mixed-effects models yielded consistent results (data not shown). Third, several factors that may influence choroidal thickness measurements were not controlled for, including axial length (AL) and circadian variations. Although patients with high myopia ( > ± 3.0D) were excluded, AL is a more precise predictor of choroidal thickness and should be measured in future studies [30]. Additionally, OCT examinations were performed during clinic hours without standardized timing, which may have introduced variability due to known diurnal fluctuations in choroidal thickness [31].

Fourth, the follow-up duration of 6 months is insufficient to evaluate long-term structural changes, sustainability of choroidal thinning, or late visual outcomes. Fifth, this study did not analyze the vascular and stromal components of the choroid separately (e.g., using choroidal vascularity index, CVI), nor did it assess systemic factors such as glycemic control (HbA1c) or blood pressure, which may confound the relationship between anti-VEGF therapy and choroidal thickness changes. Future studies incorporating CVI analysis and comprehensive systemic data collection would provide more detailed insight into the mechanisms underlying observed thickness changes.

Future research should: (1) include larger, multicenter prospective studies with long-term follow-up (≥ 2 years); (2) incorporate control groups receiving sham injections or alternative treatments; (3) measure axial length and consider its impact on choroidal thickness; (4) collect comprehensive systemic data (HbA1c, blood pressure, medications) and adjust for these confounders in multivariate analyses; (5) analyze choroidal vascularity index to differentiate vascular from stromal changes; (6) standardize OCT examination timing to minimize circadian effects; (7) investigate whether baseline choroidal thickness or early changes predict long-term visual outcomes; and (8) explore the relationship between choroidal changes and other OCT biomarkers such as hyperreflective foci and disorganization of retinal inner layers (DRIL). In subsequent research, we will continue to conduct follow-up assessments of these participants to observe long-term changes in choroidal thickness and their relationship with visual acuity recovery.

## Data Availability

Availability of data and materials Patient data are registered in medical records and on the OCT device. Data can only be accessed with express permission from Ethical Committee, patients or their legal representatives. To obtain permission to access patient and research data, should be contact the following investigator: Xiaonan SHi, e-mail: bdyzx12@163.com.

## Abbreviations

VEGF: Vascular Endothelial Growth Factor
SFCT: Subfoveal Choroidal Thickness
DME: Diabetic Macular Edema
NPDR: Non-proliferative Diabetic Retinopathy
PDR: Proliferative Diabetic Retinopathy
BCVA: Best-corrected Visual Acuity
OCT: Optical Coherence Tomography
FFA: Fundus Fluorescein Angiography
CMT: Central Macular Thickness
SFCT: Subfoveal Choroidal Thickness
anti-VEGF: Anti-vascular Endothelial Growth Factor
LSD: Least Significant Difference
DR: Diabetic Retinopathy
CI-DME: Center-involved Diabetic Macular Edema
RPE: Retinal Pigment Epithelium
NO: Nitric Oxide
AL: Axial Length
